# Precise diagnosis of Alzheimer’s disease based on sex-specific gray matter characteristics

**DOI:** 10.3389/fnins.2026.1755938

**Published:** 2026-02-06

**Authors:** Jiachen Chen, Kaiping Wang, Haoling Cao, Yongfeng Liang, Yunxia Lou, Junkang Yang, Xiangtao Lin, Yuchun Tang

**Affiliations:** 1 Key Laboratory of Experimental Teratology of the Ministry of Education, Department of Anatomy and Neurobiology, School of Basic Medical Sciences, Shandong University, Jinan, Shandong, China; 2 Shandong Key Laboratory of Mental Disorders and Intelligent Control, Shandong University, Jinan, Shandong, China; 3 Shandong Key Laboratory of Digital Human and Clinical Anatomy, Institute for Sectional Anatomy and Digital Human, Cheeloo College of Medicine, Shandong University, Jinan, Shandong, China; 4 Medical Integration and Practice Center, Cheeloo College of Medicine, Shandong University, Jinan, Shandong, China; 5 Jinan Third People's Hospital, Affiliated Jinan Third People's Hospital of Jining Medical University, Jinan, Shandong, China; 6 Qilu Hospital of Shandong University, Jinan, Shandong, China; 7 Institute of Brain and Brain-Inspired Science, Shandong University, Jinan, Shandong, China

**Keywords:** Alzheimer’s disease, gray matter, machine learning, precise diagnosis, sex-specific

## Abstract

**Introduction:**

There are notable sex differences in the gray matter of Alzheimer’s disease(AD) patients’ brains, but current evidence is insufficient to prove these differences aid diagnosis effectively.

**Methods:**

Multivariate analysis of variance was performed on the preprocessed gray matter of healthy female and healthy male groups to identify the gray matter clusters with significant intergroup differences. Subsequently, multiple machine learning models were employed to develop sex-specific diagnostic models for AD.

**Results:**

We identified 11 brain regions showing sex differences, of which 8 were sex-specific in both female and male AD patients, exhibiting significant atrophy. Graph theory analysis demonstrated that the sex-specific gray matter structural brain networks in female and male AD patients exhibited distinct network alterations. We subsequently employed five advanced machine learning algorithms to develop diagnostic models for AD based on these sex-specific gray matter clusters, resulting in a notable improvement in performance.

**Discussion:**

Sex-specific gray matter characteristics can facilitate more accurate diagnosis of AD.

## 1 Introduction

Alzheimer’s disease exhibits sex disparities, with a higher prevalence among females compared to males (Ma et al., 2024; Rajan et al., 2021). This discrepancy may be attributed to a multitude of complex physiological, pathological, and psychosocial factors, including women’s longer life expectancy, hormonal fluctuations, cardiovascular risk factors, depression, sleep quality, inadequate physical activity, social roles, and social isolation (Aggarwal and Mielke, 2023; Arenaza-Urquijo et al., 2024). These factors may be associated with distinct brain connectivity patterns (Williamson et al., 2024). Specifically, male brains tend to exhibit greater intrahemispheric connectivity, whereas female brains demonstrate more interhemispheric connections (Williamson et al., 2022). This difference in connectivity patterns may account for females’ superior performance in social cognition and males’ proficiency in sensorimotor skills and spatial information processing (Eikelboom et al., 2022). Research has shown that during the early stages of AD, the rate of gray matter atrophy in female brains is typically faster than in male brains (Saloner et al., 2024). However, males experience accelerated atrophy in later stages, potentially leading to comparable levels of atrophy over time (Joynes et al., 2024). This discrepancy may be linked to the APOE4 gene, which is more prevalent in females and increases their susceptibility to AD, resulting in more pronounced hippocampal atrophy and memory decline (Colarusso et al., 2024). In summary, sex differences in brain structure in AD involve multiple factors, including genetic predispositions and connectivity patterns. These distinctions offer valuable insights into the pathogenesis of AD and pave the way for developing targeted therapies.

In the diagnosis of AD, various machine learning models have played a crucial role by leveraging imaging data (Chang et al., 2021). Models such as logistic regression, Support Vector Machine (SVM), K-Nearest Neighbor (KNN) algorithm, neural networks, and Naive Bayes have demonstrated significant diagnostic efficacy by analyzing brain structure and functional information from neuroimaging techniques like MRI and PET (Wu et al., 2022; Johnson et al., 2014; Lyu et al., 2024). Logistic regression and SVM can identify biomarkers associated with AD, such as brain atrophy, through classification algorithms, thereby enabling early detection (Bi et al., 2018). The KNN algorithm diagnoses cases by calculating similarity to known instances based on imaging features (Yang et al., 2024). Neural network models, particularly deep learning models like Convolutional Neural Networks (CNNs), can automatically learn and extract meaningful features from large datasets, showing exceptional performance in handling complex medical imaging data (Warren and Moustafa, 2023). The Naive Bayes model applies Bayes’ theorem to combine prior knowledge with new imaging data, estimating the probability of disease presence (Wei et al., 2011). The application of these machine learning models not only enhances the accuracy and efficiency of AD diagnosis but also supports personalized treatment and disease prediction.

**Figure 1:**
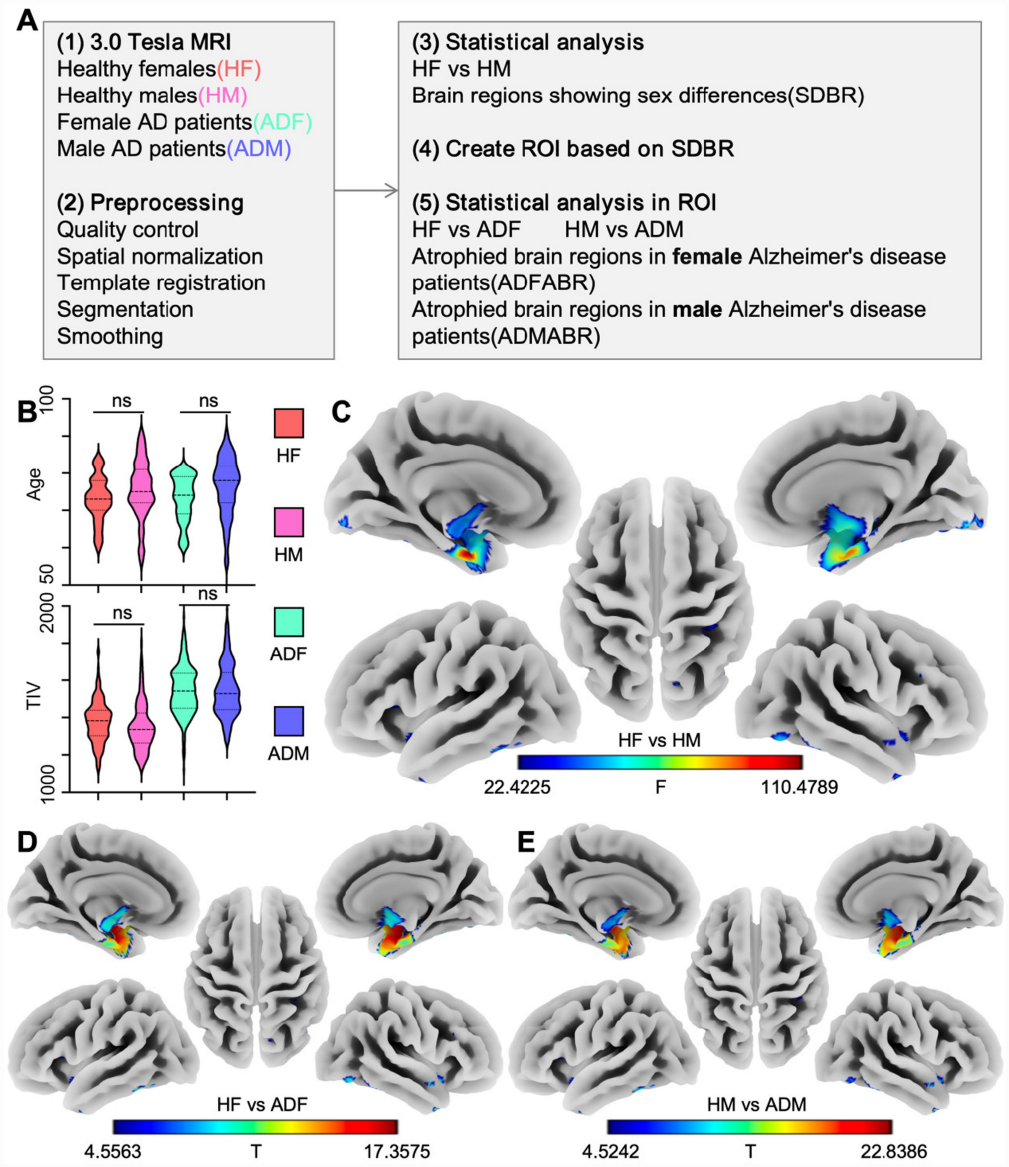
**(A)** Preprocessing and statistical analysis pipeline of brain imaging. **(B)** Statistical analysis of demographic data, *p* > 0.001. **(C)** Brain regions showing sex differences, P_FWE_ < 0.05, K > 10. **(D)** Atrophied brain regions in female AD patients, P_FWE_ < 0.05, K > 10. **(E)** Atrophied brain regions in male AD patients, P_FWE_ < 0.05, K > 10.

We aim to identify Brain Regions showing Sex Differences (SDBR) and investigate whether these regions exhibit significant atrophy in AD. Specifically, we analyze the gray matter atrophy patterns in female and male AD patients to construct sex-specific gray matter structural brain networks. Using graph theory analysis, we assess the differences in atrophy between female and male patients with AD. Finally, we utilize the sex-specific gray matter structures as input features for machine learning to develop sex-specific diagnostic models for AD (Figure 1A).

## 2 Materials and methods

### 2.1 Magnetic resonance data acquisition and preprocessing

The 3.0 Tesla structural magnetic resonance data of 164 healthy elderly female subjects and 247 healthy elderly male subjects, 135 elderly female subjects with AD and 226 elderly male subjects with AD used in our research institute all come from the ADNI database (ADNI, 2022; Table 1). First, we downsampled the object to NIFTI format images with a voxel size of 1.5 mm. Then, CAT12 was used to perform skull stripping, template registration, spatial normalization, and gray matter segmentation on the images, and an 8 mm Gaussian smoothing kernel was used to spatially smooth the gray matter (CAT, 2015). Prior to subsequent gray matter analysis, a standardized preprocessing pipeline was implemented on the original imaging data using SPM12 (Statistical Parametric Mapping 12) integrated with the CAT12 toolbox (version 12.8.0^1^), a widely validated tool for structural neuroimaging preprocessing. First, the original imaging datasets were downsampled to NIfTI-1 format images with a voxel resolution of 1.5 × 1.5 × 1.5 mm^3^. This downsampling step was performed to balance spatial resolution and computational efficiency while ensuring sufficient anatomical detail for gray matter segmentation and subsequent statistical analyses.

**Table 1:** Demographic information.

Group	Number	Age	TIV
Healthy female (HF)	164	72.9 ± 0.5	1,379 ± 9
Healthy male (HM)	247	73.1 ± 0.4	1,543 ± 8
AD female (ADF)	135	75.5 ± 0.7	1,352 ± 11
AD male (ADM)	226	76.4 ± 0.5	1,547 ± 9

Subsequently, the CAT12 toolbox was employed to conduct a series of core preprocessing steps:

1. *Skull stripping*: Non-brain tissues (including skull, scalp, and cerebrospinal fluid) were automatically removed using the built-in brain extraction algorithm of CAT12, which combines intensity thresholding and morphological operations to ensure accurate preservation of brain parenchyma.
2. *Template registration*: The skull-stripped images were linearly registered to the Montreal Neurological Institute (MNI) 152 standard template (version 2009b) to minimize inter-individual anatomical variability caused by differences in head size and shape.
3. *Spatial normalization*: Following linear registration, non-linear spatial normalization was further performed to align the individual brain images with the MNI template more precisely, which is critical for reliable inter-group comparisons of gray matter morphology.
4. *Gray matter segmentation*: Based on the normalized images, voxel-wise segmentation was conducted to separate brain tissue into three primary components (gray matter, white matter, and cerebrospinal fluid) using a hidden Markov random field model integrated in CAT12, ensuring high accuracy of gray matter tissue classification.

Finally, to reduce noise and enhance the signal-to-noise ratio (SNR) of the gray matter images—thereby improving the statistical power of subsequent group comparison analyses—an 8 mm full-width at half-maximum (FWHM) Gaussian smoothing kernel was applied to the segmented gray matter maps in three-dimensional space. All preprocessing parameters were set to CAT12 default values unless otherwise specified, ensuring reproducibility of the processing pipeline (CAT, 2015).

### 2.2 Statistical analysis

We conducted sex regression analysis on the smoothed gray matter structure of females and males using multivariate regression analysis, and during the regression process, we excluded the influences of variables such as age and total intracranial volume (Figure 1B). In our study, the scanning parameters of all the images were consistent, and the image quality scores were all greater than 75%. Therefore, other factors such as the site, scanning parameters, and image quality were not considered in the study. Similarly, when conducting paired sample T-tests on healthy subjects and subjects with AD, the influences of factors such as age and total intracranial volume were also eliminated. All image analyses require FWE correction, and a corrected *p*-value less than 0.05 and a K value greater than 10 are considered to have statistical differences.

### 2.3 Construction of sex-specific gray matter structure brain networks and graph theory analysis

We constructed the female-specific gray matter structure brain network and the male-specific gray matter structure brain network by using the Pearson correlation coefficient between sex-specific gray matter structures. A Pearson correlation coefficient greater than 0.3 and a *p*-value less than 0.05 were considered as the existence of connections between network nodes (Pan et al., 2024). Then we used the analytical method of graph theory to measure parameters such as closeness centrality, clustering nodes, eccentricity, global efficiency nodes, local efficiency nodes, path length, strength, triangles of each node in the brain network. Then, a *t*-test was conducted on healthy subjects and subjects with AD. A test p value less than 0.05 was considered to have a statistically significant difference.

### 2.4 Machine learning model

We have selected a total of five machine learning algorithms that are relatively common in the diagnosis of AD, such as logistic regression, support vector machine, K-nearest neighbor algorithm, neural network, and naive Bayes classification. All data used for constructing the machine learning classification model were randomly split into a training set and a test set at a ratio of 8:2. Our models were divided into a total of three groups. Among them, the input features of the sex-mixed AD diagnosis model are sex-related gray matter structures, the input features of the female AD diagnosis model are female-specific gray matter structures, and the input features of the male AD diagnosis model are male-specific gray matter structures. In the process of model evaluation, we selected accuracy, precision, recall rate, F1-score, AUC value, confusion matrix, ROC curve, etc. to conduct a comprehensive evaluation of the models.

## 3 Results

### 3.1 Sex-specific gray matter clusters

We performed a multivariate regression analysis on the preprocessed 3.0 T structural magnetic resonance images of healthy female and male subjects, identifying 11 brain regions showing sex differences (SDBR, Figure 1C; Tables 2, 3). The majority of these brain regions are located in the insula, with a minority distributed in the temporal and occipital lobes. Then, we utilized the 11 gray matter clusters as masks and performed paired-sample *t*-tests on the gray matter images of healthy female subjects and female subjects with AD. Our analysis revealed that 8 out of the 11 atrophied brain regions in female AD patients (ADFABR, Figure 1E; Table 4). Similarly, in the male cohort, 8 out of the 11 atrophied brain regions in male AD patients (ADMABR; Figure 1D; Table 5).

**Table 2:** Localization and description of SDBR.

Serial Number	Localization	Description
Cluster 1	Left limbic lobe	One of the core sex-related differential regions
Cluster 2	Right limbic lobe	Symmetrically distributed with Cluster 1, both are key differential regions of the limbic lobe
Cluster 3	Right occipital lobe	Main sex-related differential cluster in the occipital lobe
Cluster 4	Sub-lobar region	Sex-related gray matter structure at the sub-lobar level
Cluster 5	Left inferior temporal gyrus, temporal pole	Key functional region of the temporal lobe, involved in language and memory-related processing
Cluster 6	Left occipital lobe	Located in the left and right occipital lobes, respectively, with Cluster 3, forming a pair of sex-related differential clusters in the occipital lobe
Cluster 7	Right parietal lobe	The only clearly defined sex-related differential cluster in the parietal lobe
Cluster 8	Left occipital lobe	Supplementary sex-related differential region within the occipital lobe
Cluster 9	Right middle temporal gyrus	Core differential region in the middle of the temporal lobe, associated with auditory and memory functions
Cluster 10	Right temporal lobe	Sex-related gray matter structure in the lateral temporal lobe
Cluster 11	Right temporal lobe	Belongs to the right temporal lobe along with Cluster 10, serving as an adjacent differential region

**Table 3:** Brain regions showing sex differences (SDBR).

Serial Number	K_E_	F	P_FWE_	X	Y	Z
Cluster 1	3,408	110.48	<0.001	−16	−4	−30
Cluster 2	4,602	86.43	<0.001	20	−4	−34
Cluster 3	1,269	55.5	<0.001	3	−86	−14
Cluster 4	727	47.49	<0.001	4	0	−10
Cluster 5	81	37.58	0.005	−42	−30	−32
Cluster 6	91	35.69	0.005	−39	−88	−21
Cluster 7	165	33.9	0.001	39	−36	39
Cluster 8	252	3.82	<0.001	56	−66	−18
Cluster 9	175	33.08	0.001	36	18	−36
Cluster 10	68	31.3	0.007	32	−3	−52
Cluster 11	33	26.78	0.015	50	10	−18

**Table 4:** Atrophied brain regions in female AD patients (ADFABR).

Serial Number	K_E_	T	P_FWE_	X	Y	Z
Cluster 1	3,032	17.36	<0.001	−27	−9	−16
Cluster 2	4,069	16.96	<0.001	18	−4	−15
Cluster 9	171	11.81	0.002	36	14	−33
Cluster 5	77	10.95	0.008	−39	−28	−27
Cluster 7	128	10.10	0.004	45	−36	45
Cluster 4	581	9.76	0.01	10	−4	−12
Cluster 10	53	8.81	0.012	30	−2	−48
Cluster 8	172	8.35	0.002	56	−60	−18

**Table 5:** Atrophied brain regions in male AD patients (ADMABR).

Serial Number	K_E_	T	P_FWE_	X	Y	Z
Cluster 2	4,321	22.84	<0.001	27	−9	−16
Cluster 1	2,973	22.39	<0.001	−28	−9	−15
Cluster 9	175	13.56	0.002	36	14	−34
Cluster 5	75	12.99	0.008	−39	−28	−27
Cluster 4	450	9.56	<0.001	0	4	−8
Cluster 8	134	7.47	0.004	57	−63	−15
Cluster 7	156	7.39	0.003	40	−36	46
Cluster 6	42	5.51	0.015	−39	−86	−16

### 3.2 Sex-specific gray matter structural brain networks

**Figure 2:**
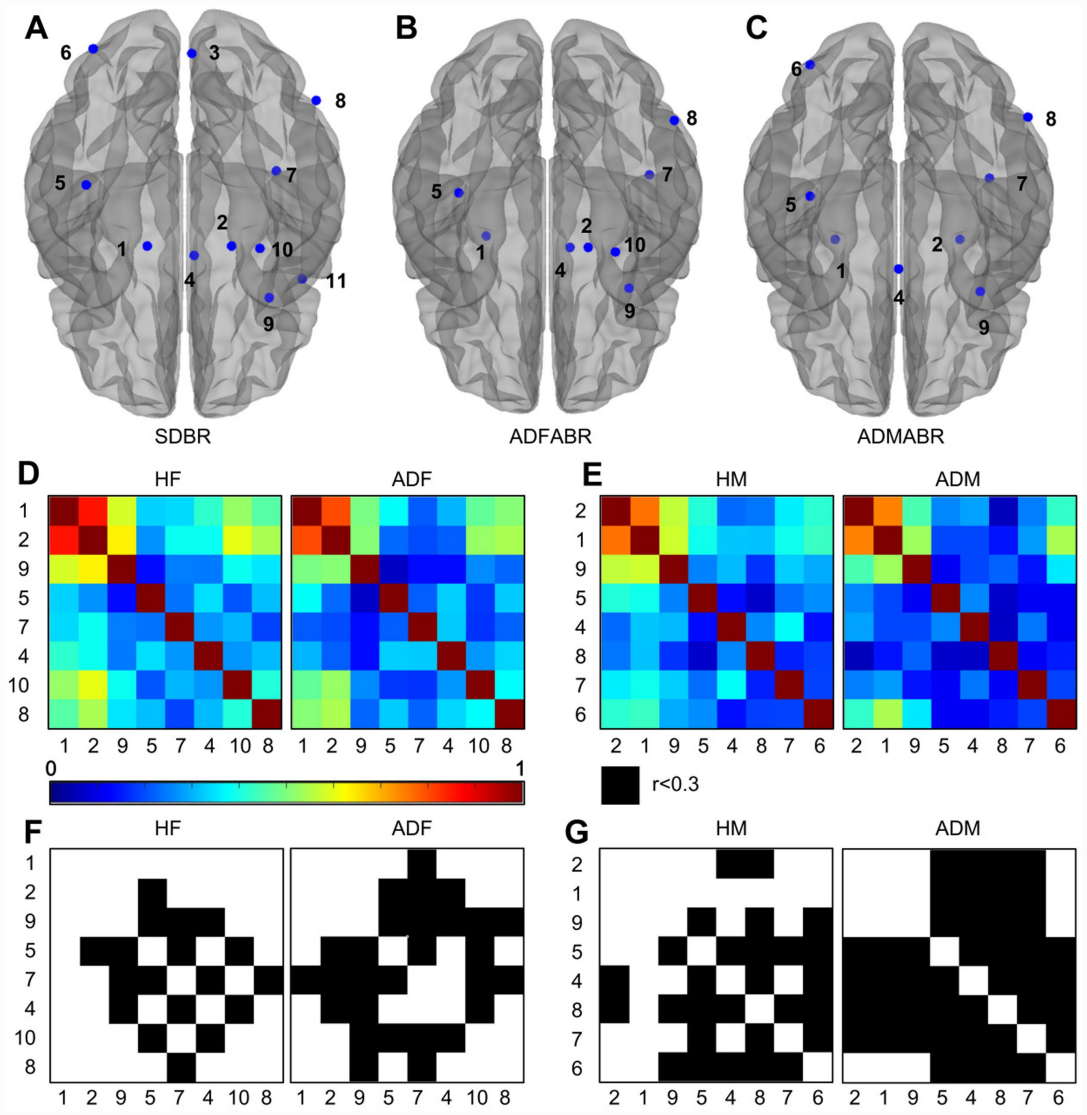
**(A)** The central coordinates of the SDBR. **(B)** The central point coordinates of the ADFABR. **(C)** The center point coordinates of the ADMABR. **(D)** The heatmap of brain network connections of ADFABR, with healthy subjects on the left and patients with AD on the right. **(E)** The heatmap of brain network connections of ADMABR, with healthy subjects on the left and patients with AD on the right. **(F)** The threshold graph of the connection strength of ADFABR network is greater than 0.3. **(G)** The threshold graph of the connection strength of ADMABR network is greater than 0.3.

Among the 11 SDBR, female Alzheimer’s patients did not exhibit significant atrophy in the 3rd, 6th, and 11th clusters (Figures 2A,B). Male Alzheimer’s patients, on the other hand, did not show significant atrophy in the 3rd, 10th, and 11th clusters (Figure 2C). Then, we acquired the absolute gray matter volumes of the eight distinct gray matter clusters for both female and male AD patients, and constructed sex-specific gray matter structural brain networks (Figures 2D,E). After setting the nodal correlation threshold of the gray matter structural brain network to 0.3, we observed a significant reduction in the overall connectivity of this network in both female and male AD patients (Figures 2F,G).

### 3.3 Graph-theoretical analysis of sex-specific gray matter structural brain networks

**Figure 3:**
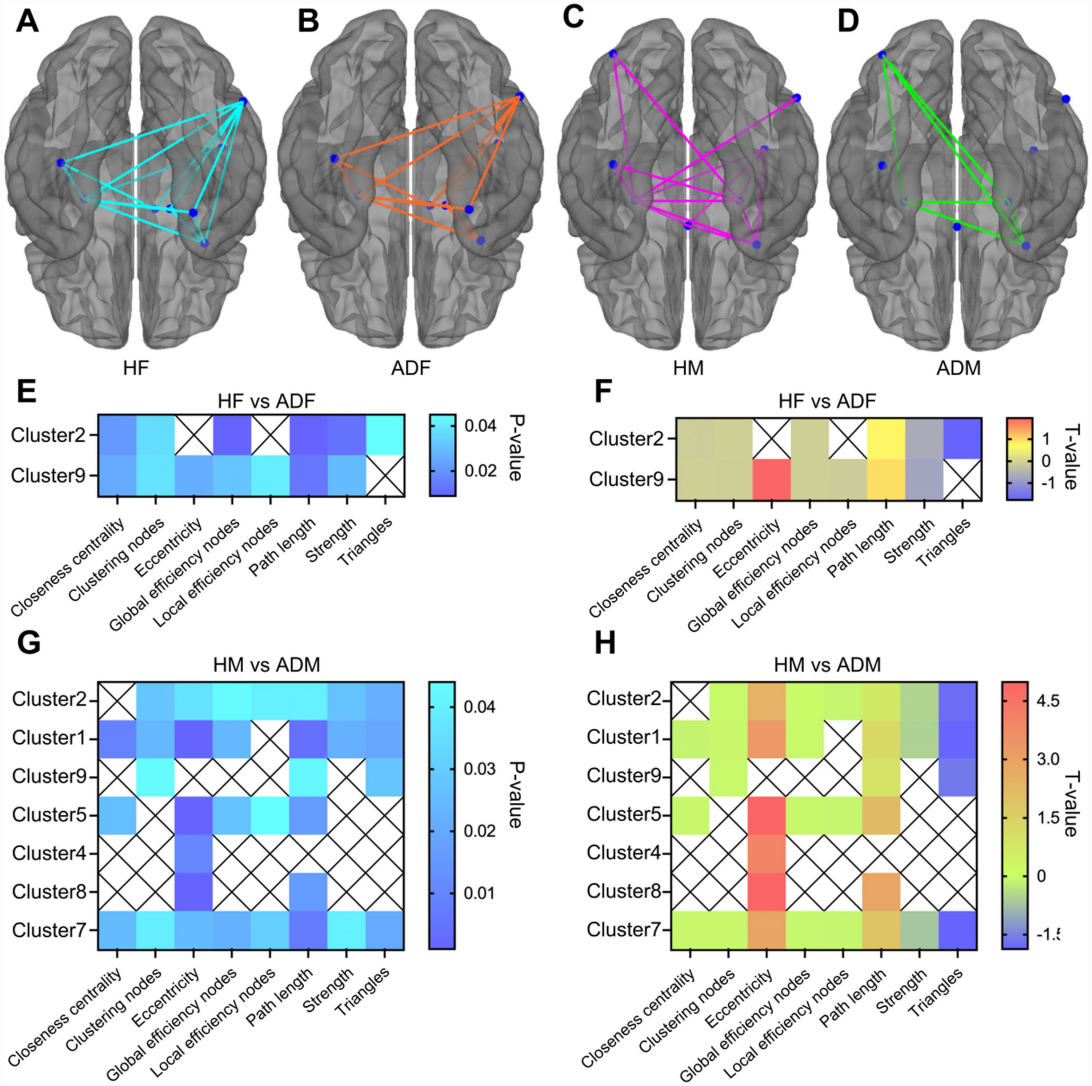
**(A)** The network connection pattern diagram of the female-specific gray matter structural brain network in healthy female subjects. **(B)** The network connection pattern diagram of the female-specific gray matter structural brain network in AD female subjects. **(C)** The network connection pattern diagram of the male-specific gray matter structural brain network in healthy male subjects. **(D)** The network connection pattern diagram of the male-specific gray matter structural brain network in AD male subjects. **(E)** The *p*-value map of the local graph theory indicators with significant changes in female AD patients. **(F)** The mean value discrepancy map of the local graph theory indicators with significant changes in female AD patients. **(G)** The *p*-value map of the local graph theory indicators with significant changes in male AD patients. **(H)** The mean value discrepancy map of the local graph theory indicators with significant changes in male AD patients. *p* < 0.05.

Figures 3A,B illustrate the connection patterns of the female-specific gray matter structural brain network in healthy females (HF) and female Alzheimer’s disease (AD) patients (ADF), respectively, while Figures 3C,D depict the corresponding patterns of the male-specific gray matter structural brain network in healthy males (HM) and male AD patients (ADM). Comparisons reveal that regardless of sex, AD patients exhibit a significant reduction in the connection complexity of their gray matter structural brain networks: the brain networks of healthy individuals show a dense and balanced connection distribution with tight associations between nodes, whereas AD patients display a marked decrease in connection density, with disrupted or weakened associations between some nodes and impaired integrity of the overall network. Notably, the reduction in network connection complexity is more pronounced in male AD patients — the brain networks of healthy males exhibit a complex pattern of multi-node collaborative associations, while those of male AD patients lose numerous connections, retaining only weak associations between a few core nodes. This magnitude of change far exceeds that observed in female AD patients.

Figure 3E (p-value map) and Figure 3F (mean difference map) clearly demonstrate the significant changes in local graph theory metrics within the female-specific gray matter structural brain network of female AD patients compared to healthy females. The results indicate that the metric alterations in female AD patients are highly localized, confined to only two gray matter clusters: Cluster 2: A total of six local graph theory metrics show statistically significant changes (*p* < 0.05), including six out of the following eight metrics: closeness centrality, clustering coefficient, eccentricity, global efficiency, local efficiency, path length, node strength, and triangle count. The mean difference map reveals that these metrics all deviate regularly from healthy levels, reflecting impaired core hub function of this cluster in the network. Cluster 9: Seven local graph theory metrics undergo significant changes, covering most of the aforementioned indicators with a mean difference magnitude similar to that of Cluster 2. This suggests that as a key node in the female-specific network, the local connection characteristics and information transmission efficiency of this cluster are significantly affected by the AD pathological process.

No significant changes in local graph theory metrics are observed in other gray matter clusters of the female-specific brain network (e.g., Clusters 1, 4, 5), indicating that the gray matter network damage in female AD patients exhibits a “locally focused” feature.

In stark contrast to female AD patients, Figure 3G (*p*-value map) and Figure 3H (mean difference map) show that the alterations in local graph theory metrics in male AD patients exhibit a “widely diffused” characteristic, involving 7 gray matter clusters (Clusters 1, 2, 4, 5, 7, 8, 9) in the male-specific gray matter structural brain network, with a total of 33 local graph theory metrics displaying statistically significant differences (*p* < 0.05): Cluster 1: Core metrics such as closeness centrality, node strength, and clustering coefficient show significant changes. The mean difference map indicates a substantial decline in node association strength and information integration capacity. Cluster 2: Similar to Cluster 2 in female AD patients, multiple metrics exhibit significant deviations but with a greater magnitude of change, accompanied by additional abnormalities in metrics such as triangle count. Clusters 4, 5, 7, 8: Each cluster shows 3–5 significant changes in local metrics, mainly concentrated in indicators reflecting network information transmission efficiency (e.g., global efficiency, local efficiency, path length), suggesting impaired functional synergy of these clusters in the male brain network. Cluster 9: As the only cluster with metric changes common to both male and female patients, male AD patients exhibit a greater number of abnormal metrics and a more pronounced mean difference magnitude in this cluster, indicating more severe damage in the male network.

Overall, the changes in local graph theory metrics in male AD patients are not only more extensive in scope but also generally show a higher degree of deviation from healthy levels compared to female patients, reflecting more comprehensive and severe damage to the gray matter structural brain network in male AD patients.

### 3.4 Sex-specific AD diagnostic model

The core difference among the three AD diagnostic models constructed in the study lies in the sex-specific design of their input parameters. Specifically, the sex-mixed AD diagnostic model takes 11 gray matter clusters with significant sex differences (SDBR) identified through multivariate regression analysis as its core input parameters. It does not distinguish between patients’ sexes or conduct sex-specific screening, covering gray matter structures in multiple brain regions such as the limbic lobe and temporal lobe as well as related quantitative imaging features, and has the largest number of input parameters among the three models. The core input parameters of the female-specific AD diagnostic model are female-specific atrophied gray matter clusters (ADFABR), which are derived from the screening of 11 SDBRs. Only 8 gray matter clusters that show significant atrophy in female AD patients compared to healthy females are retained (excluding Clusters 3, 6, and 11), covering female-specific atrophic brain regions such as the left limbic lobe and right middle temporal gyrus, and correlating with the specific brain atrophy patterns of female AD patients. The male-specific AD diagnostic model, on the other hand, uses male-specific atrophied gray matter clusters (ADMABR) as its core input parameters. Also based on the screening of 11 SDBRs, it includes 8 gray matter clusters that exhibit significant atrophy in male AD patients compared to healthy males (excluding Clusters 3, 10, and 11), encompassing male-specific atrophic brain regions such as the left limbic lobe and left occipital lobe (Cluster 6). There is partial overlap in the input parameters with the female-specific model, but there are differences in the inclusion and exclusion of sex-specific clusters. All input parameters are derived from 3.0 T structural magnetic resonance images processed by the CAT12 tool, with quantitative indicators such as gray matter volume and density extracted. Confounding factors such as age and total intracranial volume have been excluded, and the parameters have undergone FWE correction (*p* < 0.05) and voxel count screening (K > 10) to ensure statistical reliability. The sex-specific differences in input parameters enable the specialized models to reduce interference from irrelevant features and improve diagnostic accuracy.

**Figure 4:**
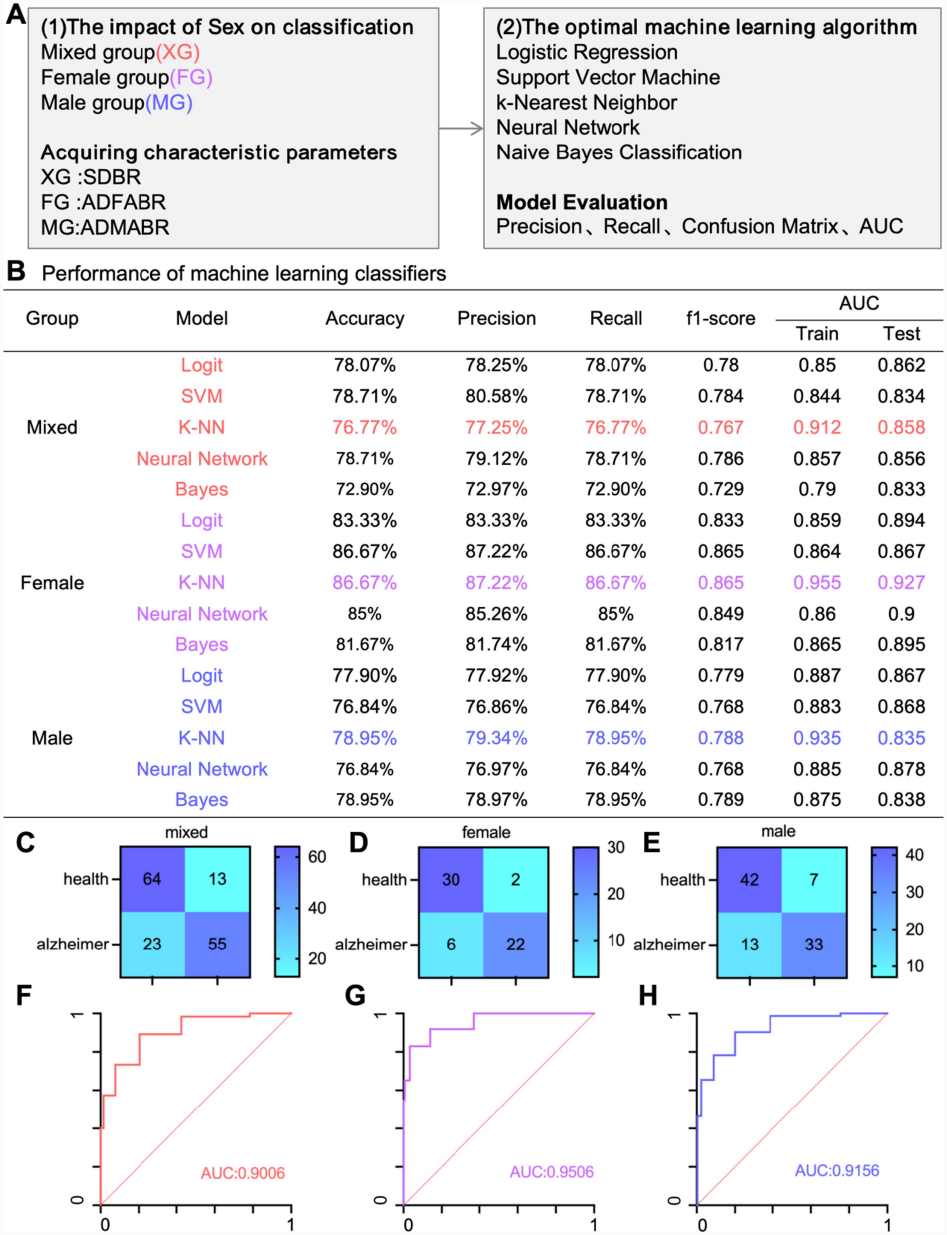
**(A)** The construction process of a sex-specific AD diagnosis model. **(B)** Summary of model evaluation for machine learning algorithms. **(C)** The confusion matrix of the sex-mixed AD diagnosis model for diagnosing the validation set. **(D)** The confusion matrix of the female-specific AD diagnosis model for diagnosing the validation set. **(E)** The confusion matrix of the male-specific AD diagnosis model for diagnosing the validation set. **(F)** The ROC curve of the sex-mixed AD diagnosis model for all the subjects. **(G)** The ROC curve of the female-specific AD diagnosis model for all the subjects. **(H)** The ROC curve of the male-specific AD diagnosis model for all the subjects.

We employed five machine learning algorithms (Logistic Regression, Support Vector Machine, K-Nearest Neighbor, Neural Network, and Naive Bayes) to develop three distinct AD diagnostic models: a mixed-sex model, a female-specific model, and a male-specific model (Figure 4A). The diagnostic performance of these models was assessed using accuracy, precision, recall, F1 score, and AUC (Figure 4B). Combining five evaluation indicators, we found that despite having the highest number of input feature values, the mixed-sex model exhibited the lowest diagnostic performance (AUC 0.9006; Figures 4C,F). In contrast, both the female-specific AD diagnostic model (AUC 0.9506; Figures 4D,G) and the male-specific AD diagnostic model (AUC 0.9156; Figures 4E,H) demonstrated relatively high diagnostic performance. Among the five machine learning models evaluated, the K-nearest neighbor algorithm demonstrated superior diagnostic performance. These findings suggest that leveraging sex-specific gray matter structures for diagnosing AD can achieve notably high diagnostic accuracy.

## 4 Discussion

We performed multivariate regression analysis on preprocessed 3.0 T structural magnetic resonance images of healthy elderly females and males, which identified 11 gray matter clusters with significant sex associations. Specifically, the first and second clusters were predominantly localized to the left and right limbic lobes, respectively. The third, sixth, and eighth clusters were primarily distributed in the right and left occipital lobes. The fourth cluster was mainly situated in the sub-lobar region, while the fifth cluster was concentrated in the left inferior temporal gyrus and temporal pole. The seventh cluster was primarily located in the right parietal lobe, and the ninth cluster was centered in the right middle temporal gyrus. Notably, both the tenth and eleventh clusters were found in the right temporal lobe. Collectively, these sex-related brain regions are predominantly concentrated in the limbic lobe and temporal lobe.

Extensive research has been conducted on sex differences in the limbic lobe and temporal lobe, yielding substantial advancements (Gur et al., 2002; Mejías-Aponte et al., 2002). Studies have consistently shown that brain structures in these regions are generally larger in males than in females, with the temporal lobe exhibiting particularly prominent sexual dimorphism (Kawano et al., 2024). This structural discrepancy is hypothesized to underpin the observed variations in cognitive and behavioral traits between the sexes (Poole et al., 2024; Zhang et al., 2024). For example, males often demonstrate superior performance in spatial orientation, mathematical reasoning, and mechanical skills, whereas females tend to excel in language processing, social cognition, and emotional regulation tasks (Chen et al., 2020; Benbow and Stanley, 1983; Streamer and Queen, 2024; Sato, 2020; Paletta et al., 2023). These sex-specific differences in brain structure are believed to arise from the intricate interplay of genetic factors, hormonal influences, and socio-cultural environments.

Among the 11 gray matter clusters, several clusters exhibit significant atrophy in both female and male AD patients, specifically the 1st, 2nd, 4th, 5th, 7th, 8th, and 9th clusters. However, the extent of atrophy varies between these clusters. Additionally, female AD patients show significant atrophy specifically in the 10th cluster, whereas male AD patients exhibit significant atrophy in the 6th cluster. Despite the relatively small volumes of these two gray matter clusters, the stability of each node within a network is fundamental to maintaining the overall network stability. We constructed sex-specific gray matter structural brain networks for females and males respectively, based on sex-specific gray matter clusters. We compared the gray matter structural brain networks of the healthy group and the AD group and found that the complexity of the gray matter structural brain network connections in both female and male AD groups decreased significantly. Studies have demonstrated that, in comparison to healthy brains, AD patients exhibit increased brain network path lengths and decreased global efficiency, indicative of significant structural damage (Moqri et al., 2023; Cassady et al., 2023). This damage is closely associated with the pathological hallmarks of AD, including amyloid plaques, neurofibrillary tangles, neuronal loss, and synaptic dysfunction (Soreq et al., 2023; Gerrits et al., 2021).

Although both the sex-specific gray matter structural brain networks of female and male AD patients exhibit a similar downward trend in complexity, the alterations in network nodes differ markedly. Using graph theory analysis, we observed that in the female-specific gray matter structural brain network, significant changes were primarily confined to closeness centrality, clustering coefficient, path length, and node strength within the 2nd and 9th clusters. In contrast, the male-specific gray matter structural brain network demonstrated significant differences across seven clusters when comparing male AD patients with healthy controls. We observed that the alterations in the sex-specific gray matter structural brain network were more pronounced in male patients compared to female patients. Furthermore, research has demonstrated that while both male and female patients exhibit abnormal brain network connections and gray matter atrophy, the changes in brain structure among male patients possess distinct characteristics (Beckett, 2022; Li et al., 2021). Although the rate of atrophy in certain brain regions may be relatively slower in males compared to females, particularly in the early stages of the disease, male patients are more prone to functional abnormalities in specific brain networks, such as those linking the visual and motor cortices (Park et al., 2019). These sex differences could be attributed to physiological traits, genetic backgrounds, and lifestyle factors unique to men (Eissman et al., 2024; Podcasy and Epperson, 2016). Additionally, studies have indicated that male patients may respond differently to certain AD treatment drugs compared to females, underscoring the significance of incorporating sex considerations in the diagnosis and treatment of AD (Lynch, 2024; Canevelli et al., 2017). Consequently, personalized treatment plans for male and female patients should take into account their respective sex-specific characteristics to enhance therapeutic outcomes.

We developed three distinct AD diagnosis models: a mixed-sex model utilizing sex-related gray matter structures, a female-specific model using female-specific gray matter structures, and a male-specific model using male-specific gray matter structures. Model evaluation revealed that the diagnostic accuracy of the sex-separated models was superior despite having fewer input features compared to the mixed-sex model. Our study employed five machine learning algorithms—logistic regression, support vector machine (SVM), K-nearest neighbor (KNN) algorithm, neural network, and naive Bayes. The KNN algorithm demonstrated the highest diagnostic performance. Previous studies have extensively reported on SVM and random forest algorithms (Wang et al., 2023; Sarica et al., 2017), but our findings highlight the effectiveness of KNN In the context of sex-specific AD diagnosis. In sex-specific AD (AD) diagnosis, the KNN algorithm can effectively leverage patient sex and imaging biomarkers to enhance diagnostic performance (Elgammal et al., 2022). The simplicity and ease of implementation of the KNN algorithm allow it to operate without extensive data preprocessing, offering significant flexibility in clinical settings. However, the KNN algorithm has limitations, including sensitivity to data distribution and class imbalance, as well as high computational demands. In the context of sex-specific AD diagnosis, these issues may result in diagnostic inaccuracies or reduced computational efficiency. Therefore, while the KNN algorithm holds potential value for sex-specific AD diagnosis, further research and optimization are necessary to improve its diagnostic accuracy and computational efficiency.

## Data Availability

The datasets presented in this study can be found in online repositories. The names of the repository/repositories and accession number(s) can be found at: https://adni.loni.usc.edu/.

## Footnotes

1
https://www.neuro.uni-jena.de/cat12/
